# Overnutrition-induced gout: An immune response to NLRP3 inflammasome dysregulation by XOD activity increased in quail

**DOI:** 10.3389/fimmu.2022.1074867

**Published:** 2022-12-08

**Authors:** Hao Wu, Yu Wang, Zhixin Ren, Yaolei Li, Jingjian Huang, Zhijian Lin, Bing Zhang

**Affiliations:** ^1^ Department of Clinical Chinese Pharmacy, School of Chinese Materia Medica, Beijing University of Chinese Medicine, Beijing, China; ^2^ Center for Pharmacovigilance and Rational Use of Chinese Medicine, Beijing University of Chinese Medicine, Beijing, China

**Keywords:** overnutrition, gout, immune response, NLRP3 inflammasome, XOD

## Abstract

**Background:**

Gout is a progressive metabolic disease closely related to hyperuricemia and urate deposition, with an increasing prevalence and incidence across the globe. Recent studies have shown that the pathological process of gout includes two stages: asymptomatic hyperuricemia and MSU crystal deposition. However, the immune response during the development of hyperuricemia to gouty arthritis is not fully elucidated.

**Methods:**

Thus, an overnutrition-induced whole-course gout model was established to clarify the immune response and pathological changes in the development from hyperuricemia to gouty arthritis. The quails without urate oxidase were used as experimental animals. And we confirmed that uric acid metabolic targets were changed when quails were in the asymptomatic hyperuricemia stage.

**Results:**

When the quail showed gout symptoms, the NLRP3 inflammasome was activated, and the expressions of IL-1β, TNF-α, IL-6, IL-8, and IL-18 were significantly increased. The relationship between the uric acid metabolism target and the NLRP3 inflammasome may be the critical immune response between hyperuricemia and gouty arthritis. Our data showed that, in the process of gout disease, the expression of xanthine oxidase (XOD) has been increasing, which increases the level of uric acid, disrupts the balance of oxidative stress, generates a large amount of ROS, activates the NLRP3 inflammasome, and release IL-1β. Treatment with the XOD inhibitor can reduce uric acid, restore the body’s degree of peroxidative damage and antioxidant capacity, and inhibit NLRP3 inflammasome and IL-1β. *In vitro*, we extracted and identified primary fibroblast-like synoviocytes (FLS) from quail for the first time. Stimulating FLS with uric acid also caused ROS release and NLRP3 inflammasome activation. However, treatment with an XOD inhibitor prevented all these responses in FLS.

**Conclusion:**

Our results indicate that the immune response between the uric acid metabolism target XOD and NLRP3 inflammasomes plays a crucial role in developing hyperuricemia to gouty arthritis, and inhibition of both XOD and NLRP3 inflammasomes may be an effective treatment for avoiding the development of asymptomatic hyperuricemia to MSU crystal deposition. Meanwhile, this study also provides an advantageous animal model for pathological mechanisms and research and development drugs for gout.

## Introduction

Gout is a metabolic disease affected by monosodium urate (MSU) deposition, which is directly related to hyperuricemia caused by purine metabolism disorders and/or decreased uric acid excretion. It results from the testimony of urate crystals in joints, tendons, and other tissues caused by abnormally elevated levels of circulating urate and related to overnutrition (1, 2). Presently, gout also shows a trend of high incidence among younger people worldwide (3, 4). It can cause considerable disability, health loss, and economic burden, and there is an urgent need for clinical treatment (5–7).

The disease process of gout includes the hyperuricemia stage, monosodium urate (MSU) crystal deposition stage, and acute inflammation stage. It is a metabolic disease with progressive and recurrent characteristics (8, 9). 2018 updated European League Against Rheumatism evidence-based recommendations for the diagnosis of gout divide the overall clinical manifestations of gout into four stages: asymptomatic hyperuricemia stage (without MSU crystal deposition), asymptomatic MSU crystal deposition stage (without gouty arthritis attack), gouty arthritis attack and the interictal period (with MSU crystal deposition), progressive/chronic gouty arthritis period (tophi, bone destruction) (10). In 2019, Gout, Hyperuricemia, and Crystal-Associated Disease Network (G-CAN) defined hyperuricemia and gout as distinct states of the same disease (11). In 2020, China’s Guidelines for the Diagnosis and Treatment of Hyperuricemia and Gout (2019), advanced imaging examination newly found urate deposition in the joints and tissues of some asymptomatic hyperuricemia patients, which confirmed the continuous pathological process of hyperuricemia and gout and proposed that asymptomatic hyperuricemia with uricemia deposition is a new concept of “subclinical gout” (12). Our research group summarized the latest studies on gout worldwide and confirmed that hyperuricemia and gouty arthritis are different pathological stages of gout (13). We can see that the latest research has a new understanding of gout and gradually realizes that asymptomatic hyperuricemia and gouty arthritis are a continuous pathological process, elevated blood uric acid and inflammatory responses are the cardinal symptoms of gout.

In the past, it was generally believed that the inflammatory response only existed in the urate deposition stage of gouty arthritis. It resulted from uric acid crystals stimulating the body’s innate immune response (14, 15). Its pathological mechanism mainly involves the release of interleukin-1β (IL-1β) mediated by the NOD-like receptor thermal protein domain associated protein 3 (NLRP3) inflammasome (16, 17). Among them, the NLRP3/IL-1β inflammatory signaling pathway plays an essential role in the inflammatory process caused by urate deposition (18, 19). The NLRP3 inflammasome is composed of NLRP3, apoptosis-related speck-like protein ASC, and Caspase-1 and is a crucial pathway for splicing and releasing IL-1β (20, 21). Therefore, although the role of the NLRP3/IL-1β inflammatory signaling pathway in gout arthritis has been identified, its role in the overall progression of gout disease is unclear. Thus, an overnutrition-induced whole-course gout model was established to clarify the immune response and pathological changes in the development from hyperuricemia to gouty arthritis.

The formation of hyperuricemia is related to abnormal uric acid homeostasis, and its pathological process is regulated by the target of uric acid metabolism (22, 23). Recent studies have found that the target uric acid metabolism is closely related to an inflammatory response and regulating uric acid levels (24–28). Xanthine oxidase (XOD) is the crucial enzyme responsible for the production of uric acid in the target group of uric acid metabolism. Many studies have shown that increased XOD activity can release oxygen free radicals and promote ROS expression. When the XOD inhibitor is given for uric acid-lowering treatment, a significant decrease in serum IL-1β levels in patients with hyperuricemia can be observed (29). Uric acid is the end product of purine metabolism, and the physiological concentration of uric acid is an essential antioxidant in the body, which can reduce the oxidative damage caused by ROS (30, 31). Uric acid above the physiological concentration is mainly deposited in the body tissues or organs in the form of crystals, and is recognized as a dangerous signal, which induces mitochondrial damage, promotes the release of ROS expression, activates signaling pathways such as NLRP3 inflammasome, and causes tissue and organ damage (32, 33).

The uric acid metabolism of quail is similar to that of humans, and there is no urate oxidase in the body, which cannot convert uric acid into allantoin (34). Our previous study investigated gout pathology and drug treatment in quail (35–40). This study clarified the immune response and pathological association of XOD and NLRP3 inflammasome in gout. At the same time, fibroblast-like synoviocytes (FLS) were extracted and identified as quail for the first time. The immune response during the development of hyperuricemia to gouty arthritis may be due to the increased expression of XOD, which promotes the production of uric acid, activates the release of ROS, and then activates the NLRP3 inflammasome and release IL-1β. The immune response between uric acid metabolism targets XOD and NLRP3 inflammasomes and plays a crucial role in developing treatments for overnutrition-induced gout.

## Materials and methods

### Antibodies and reagents

Antibodies to NLRP3 (27458-1-AP) and GAPDH were purchased from Proteintech (Rosemont, IL, USA). Antibodies to vimentin were purchased from Bioss (Beijing, China). Yeast powder (LP0021B) was purchased from Oxoid (Basingstoke, United Kingdom). D (–)-Fructose (18G3056118) was purchased from Amresco (Solon, OH). Reagent test kits of IL-1β, IL-6, IL-18, TNF-α, IL-8, ASC, and Caspase-1 were purchased from Jianglai (Shanghai, China). The reagents for detecting MDA, GSH, SOD, and DCFH-DA were purchased from Solarbio (Beijing, China). Collagen I (sc-28654) was purchased from Santa Cruz Biotechnology (San Diego, CA, USA). The reagents for the detection of serum and fecal uric acid (UA), serum creatinine (Cre), and serum urea nitrogen (BUN) test kits were purchased from Jiancheng Biological Technology, Co., Ltd (Nanjing, China). DMSO and all other chemicals were obtained from Sigma-Aldrich (St. Louis, MO, USA).

### Gout quail model

All quail operations were approved by the Animal Care and Ethics Committee at the Beijing University of Chinese medicine (NO. BUCM-4-2021070102-3105). Four-week-old male French quails (Beijing Deling Quail Farm) were housed in 90 × 80 × 40 cm^3^ cages in a temperature-controlled room (temperature 25 ± 2°C, air humidity 50–55%, and 12 h light cycles). And ventilating equipment is all in the standard environment. After adaptive rearing, male French quails (n = 100, 4 weeks old) were freely provided feed formulation and water during the experimental period. Subsequently, quails were randomly divided into two groups (50 quails in each group): the control group (Con), quails were fed with a regular diet, the model group (Mod), feed quails an overnutrition diet supplemented with yeast and bone extract powder and drinking water containing 10% fructose (15ml per quail) (Patent No.202210651513.3). Feed was added twice daily, and the remainder was weighed the following day. At the same time, the daily weight of each quail was also considered and recorded. Every ten days of the experiment, fecal samples (including urine and fecal mixture) were collected in metabolic cages. After fasting for 12 h, jugular vein blood samples were collected. The samples were placed at room temperature until the serum was precipitated and centrifuged at 3500 r/min for 10 min to detect uric acid levels. On the experiment’s 10th, 20th, and 30th days, the quails of each group were collected, the joint cavity of the quails was washed with 0.2ml of normal saline, and the ankle joint fluid of each group was gathered. And the level of uric acid in the joint fluid of each group was detected.

After 30 days of the experiment, the Con group was fed typically, the Mod group quails were divided into two groups (10 quails in each group), and one group was treated with allopurinol (35 mg/kg) for ten days. After the experiment, all animals were sacrificed, the kidney and joint synovial tissue of each group of quail were collected, and some tissues were homogenized with a tissue freezer grinder for the following experiments.

### Assessment of ankle edema

Ankle edema formation was assessed as an increase in ankle circumference. We measured ankle circumference at 0.5 mm below the ankle joint on days 10, 20, and 30 of the experiment. Each measurement was repeated three times to obtain an average value. ASD was calculated using Equation (3).


$$\begin{array}{l} \text{Ankle swelling degree }\left(\%\right)\;=\;\left[\begin{array}{l} \text{Ankle circumference after modeling }\left(\text{mm}\right)\;-\text{ Ankle }\\ \text{circumference before modeling }\left(\text{mm}\right) \end{array}\right]/\\ \left[\text{Ankle circumference before modeling }\left(\text{mm}\right)\right]\;\times 100\%. \end{array}$$

### Measurement of biochemical indicators in gout quails

Blood samples and tissues were collected according to the experimental design every ten days. The serum and tissues were separated for measurement of SOD, MDA, GSH, XOD, 5’-NT, and ADA using ELISA kits according to its instructions.

### Acquisition and culture of primary fibroblast-like synoviocytes

In this study, fibroblast-like synoviocytes (FLS) were extracted and identified in quail for the first time. The bilateral posterior knee joints of the quail were taken. After washing the knee joints, the knee joints were transferred to a PBS plate containing 100 μ/mL penicillin and 100 mg/mL streptomycin on an ultra-clean workbench for synovial tissue separation. Isolation conditions: ice bath, sterile. Cut the synovial tissue to 1mm × 1mm × 1mm, transfer it to a 10 mL clean centrifuge tube, and centrifuge at low speed briefly; discard the supernatant, add 1 mL of 0.2% type I collagenase to resuspend the synovial tissue, and transfer it to 25 cm^2^ culture flask, dilute the volume to 3 mL with 0.2% collagenase type I, shake the culture flask gently to make the synovial tissue at the bottom evenly distributed. The synovial tissue was cultured in a 37°C, 5% CO_2_ incubator, and the morphology and growth state of synovial cells were observed under an inverted microscope every day. The medium was changed every 2-3 days.

### Immunofluorescence staining

For the third generation of synovial cells, the cell ratio was adjusted to 4 × 10^4^/mL, and the cells were cultured in an incubator for 24 hours. Add 300uL of 4% paraformaldehyde, fix for 20min, and wash 2-3 times with PBS. After permeabilization with 0.2% TritonX-100 at 37°C for 20min, add Vimentin antibody (1:200), incubate overnight at 4C in a wet box, add fluorescent 488 IgG (1:500), and incubate at 37°C for one h in the dark. After nuclei were stained with DAPI (37°C) for 15 min, the slides were mounted with an anti-fluorescence quencher. Fluorescence microscopy was observed and photographed. According to the Vimentin staining in the synovial cells tested, the purity of FLS was judged. For example, more than 98% of the cells stained with Vimentin in synovial cells, that is, more than 98% of the cells are FLS.

### Cell treatment

FLS was starved in a DMEM medium containing 0.5% fetal bovine serum for 24 hours, and then uric acid (UA, 600 mM) and allopurinol (ALP, 200 mM) were added for 24 h. After 24h of stimulation, cells were harvested for further analysis. All of the *in vitro* experiments were repeated no less than three times.

### Immunoblot analysis

Cells were washed three times with ice-cold PBS and collected in RIPA buffer containing PMSF and phosphatase inhibitors. Synovial tissue samples were homogenized with cell lysis buffer, PMSF, and phosphatase inhibitors. Proteins were separated by SDS-PAGE and transferred to 0.2 mm nitrocellulose membranes. After incubation with 5% skims milk for 1 hour at room temperature, the cells were incubated with primary antibodies overnight at 4°C and then incubated with an appropriate amount of horseradish peroxidase-conjugated secondary antibodies for 1 hour at room temperature. Bound antibodies were detected by chemiluminescence. Densitometric analysis of immunoblot results was performed using Image J software (National Institutes of Health, Bethesda, MD).

### Hematoxylin and eosin staining analysis of joint and kidney

Kidney and joint tissues were harvested and fixed in 4% polyformaldehyde at room temperature for 24 h, processed routinely for histology, and embedded in paraffin. Each specimen was cut into 5μm slices on a rotary microtome. Each section was deparaffinized in xylene, rehydrated in reduced ethanol concentrations, and stained with hematoxylin and eosin reagents.

### Statistical analysis

Data are presented in the graph as mean ± SEM for each group. All results were tested for normality to determine the use of parametric or nonparametric tests. Data containing two groups of normal distributions were analyzed with the unpaired Student’s t-test and nonparametric data with the Mann-Whitney U test. When more than two factors were present in the analysis, the Two-way ANOVA test was performed, followed by Sidak’s multiple comparison test. Statistically significant differences between mean values were marked in each graph. *P*<0.05 was considered statistically significant.

## Results

### The general state of quail

**Figure 1:**
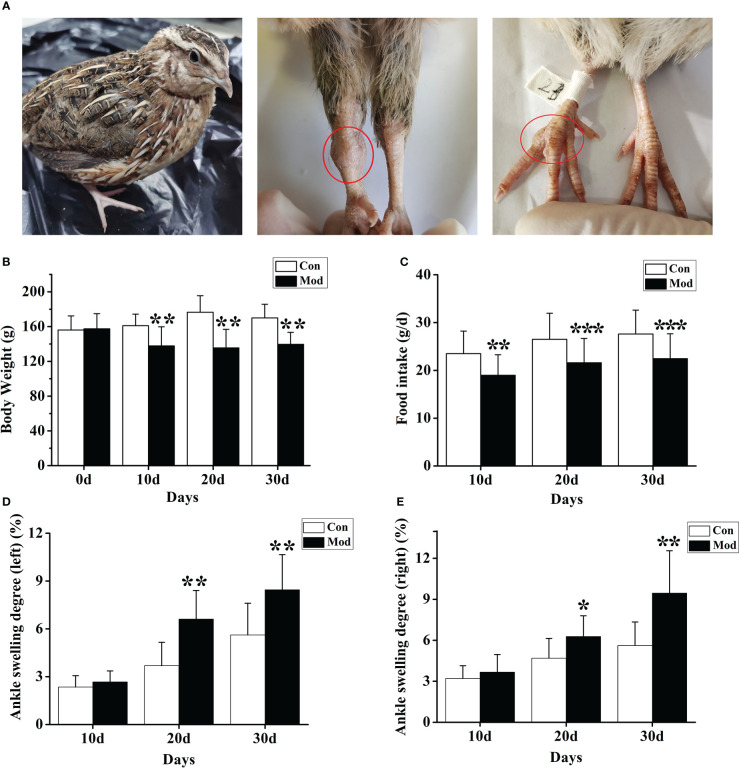
The general state of quail in the modeling process. **(A)** Swelling degree of quail joints in Mod group. **(B)** The body weight of quails in each group at different experimental stages. **(C)** The food intake of quail in each group at different experimental stages. The left **(D)** and right **(E)** of the swelling degree of the quail ankle joint during the experiment. Data are expressed as mean ± SEM for 8 quails in each group. *P < 0.05, **P < 0.01 and ***P < 0.001 versus Con group.

During the experiment, the quails in the Con group were in a normal mental state, with bright feathers, loud calls, and much activity; the quails in the Mod group were in a poor mental state, with black feathers, hoarse calls, and less movement. And with the prolongation of modeling time, the quail joints in the Mod group showed noticeable swelling, which was consistent with the clinical characteristics of gouty joints (**Figure 1A**). Compared with the Con group, the body weight level of the quails in the Mod group was significantly lower in the experiment (*P*< 0.05 or *P*< 0.01) (**Figure 1B**). The food intake levels of the quails in the Mod group were all significantly lower (*P*< 0.01 or *P*< 0.001) (**Figure 1C**). Compared with the Con group, on the 20th and 30th days of the experiment, the ankle joint swelling of the Mod group was significantly increased (*P*<0.05) (**Figures 1D, E**), indicating that with the extension of the modeling time, the Mod group quail appeared gout symptoms.

### Activation of the NLRP3 inflammasome

**Figure 2:**
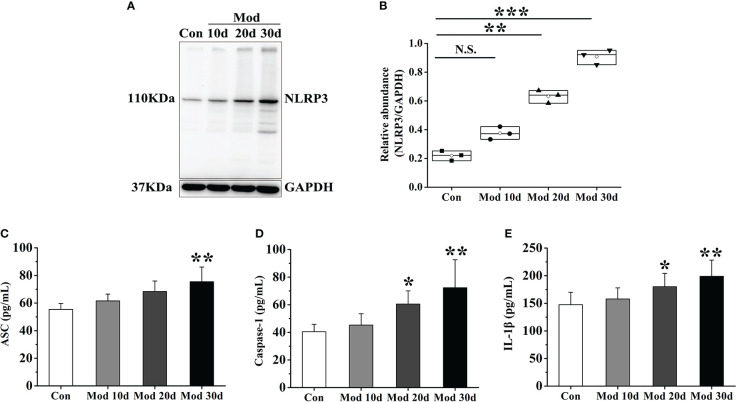
As the experiment progressed, the NLRP3 inflammasome was activated and IL-1β expression increased in synovial tissue. **(A)** Western blot was conducted to evaluate the protein level of NLRP3 and GAPDH in synovial tissue (n=3). **(B)** Box plot showing the densitometry analysis of NLRP3 normalized by GAPDH. **(C)** The expression level of ASC in synovial tissue. **(D)** The expression level of Caspase-1 in synovial tissue. **(E)** The expression level of IL-1β in synovial tissue. Data are expressed as mean ± SEM for 8 quails in each group. *P < 0.05, **P < 0.01 and ***P < 0.001 versus Con group.

After the experiment, the expressions of NLRP3, ASC, Caspase-1, and IL-1β in the synovial tissue were detected. The results showed that with the development of the experiment, the longer the modeling time was, the higher the expression level of NLRP3 in the synovial tissue. On the 20th day of the investigation, the expression of NLRP3 in the Mod group was significantly higher than that in the Con group (*P*<0.01). Compared with the Con group, the expression of NLRP3 was significantly increased (*P*<0.001) (**Figures 2A, B**). The expression levels of ASC, Caspase-1, and IL-1β in each group’s synovial tissue of quails were analyzed. On the 30th day of the experiment, the expression level of ASC in the Mod group was significantly higher than that in the Con group (*P*<0.01) (**Figure 2C**). On the 20th day and 30th day of the experiment, the expression level of Caspase-1 in the Mod group was significantly higher than that in the Con group (*P*<0.05), and on the 30th day of the experiment, the expression level of Caspase-1 in the Mod group was significantly higher than that in the Con group (*P*<0.01) (**Figure 2D**). On the 20th day, the expression level of IL-1β in the Mod group was significantly higher than that in the Con group (*P*<0.05), and on the 30th day of the experiment, the expression level of IL-1β in the Mod group was significantly higher than that in the Con group (*P*<0.01) (**Figure 2E**).

### Expression levels of uric acid and related metabolic enzymes

**Figure 3:**
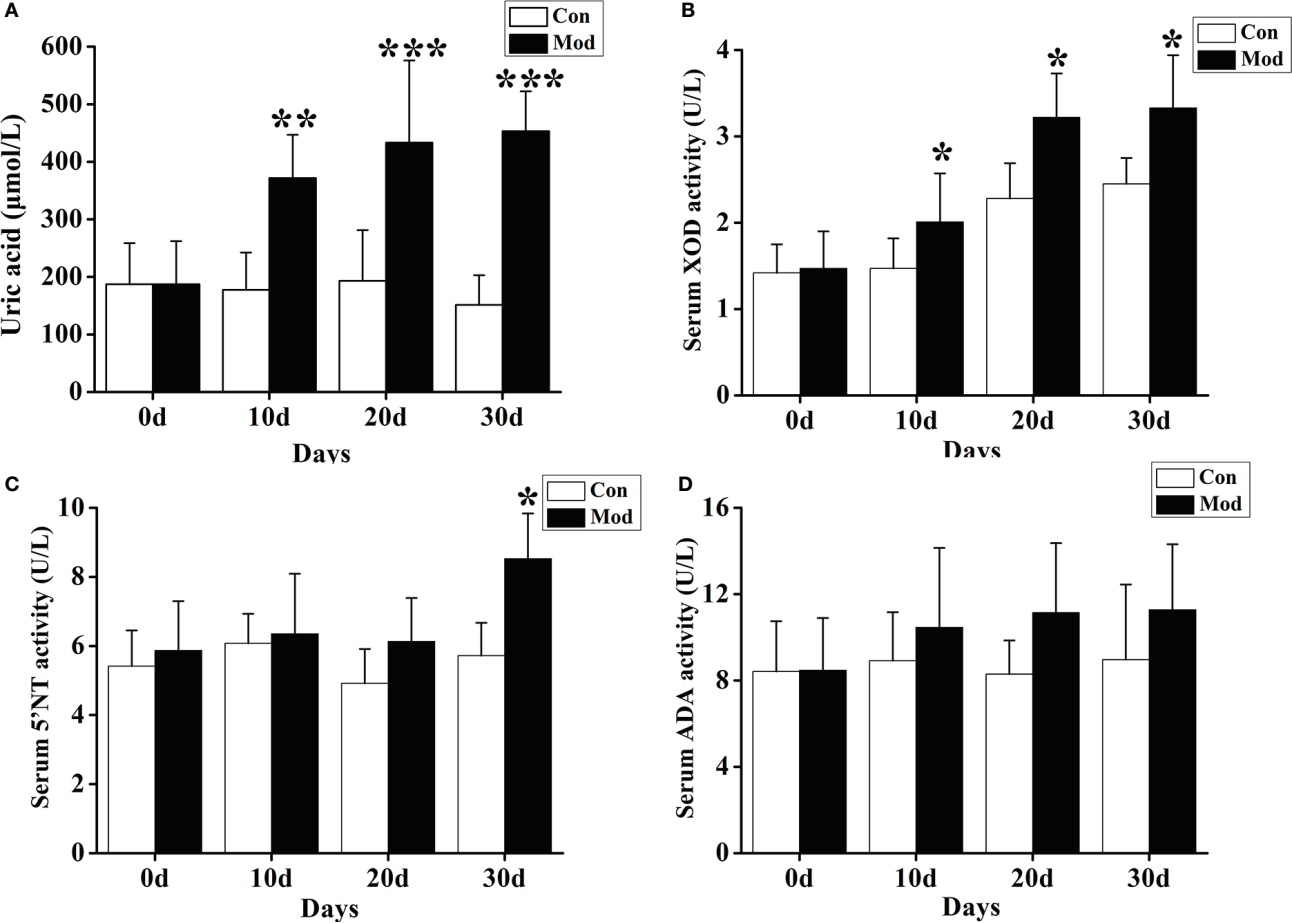
Increased uric acid level and expression of related uric acid production and metabolism enzymes. **(A)** The serum uric acid level. **(B)** The XOD activity in the serum. **(C)** The 5’-NT activity in the serum. **(D)** The ADA activity in the serum. Data are expressed as mean ± SEM for 8 quails in each group. *P < 0.05, **P < 0.01 and ***P < 0.001 versus Con group.

The results showed that compared with the Con group, the serum uric acid level of the quails in the Mod group was significantly increased on the 10th, 20th, and 30th days of the experiment (*P*< 0.01 or *P*< 0.001) (**Figure 3A**). We also tested the fecal uric acid level of quails in each group and found that the fecal uric acid level of the Mod group was higher than that of the Con group, but there was no significant change (*P* > 0.05).

Compared with the Con group, the serum XOD activity of the quails in the Mod group was significantly increased at 10, 20, and 30 days of the experiment (*P*< 0.05) (**Figure 3B**). On the 30th day of the experiment, the serum 5’-nucleotidase (5’-NT) activity of the quails in the Mod group was significantly higher than that in the Con group (*P*< 0.05). Compared with the Con group, on the experiment’s 10th, 20th, and 30th days, the serum adenine deaminase (ADA) activity of the quails in the Mod group increased. Still, there was no significant difference (*P* > 0.05) (**Figures 3C, D**). The results showed that the increase of XOD may be the main reason for the rise of uric acid in quails in the Mod group.

### Cytokine expression levels

**Figure 4:**
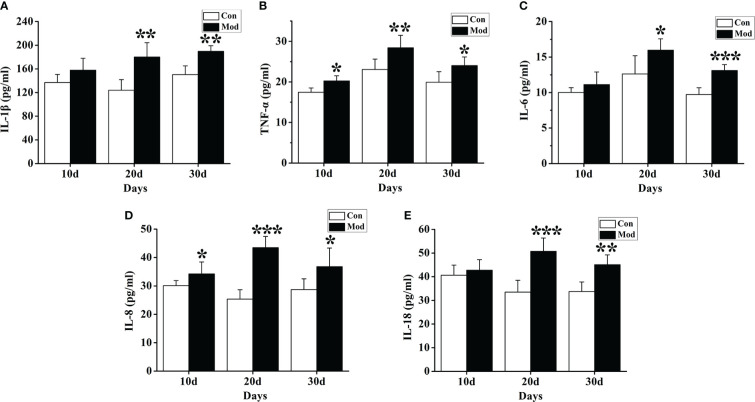
Expression of inflammatory factors in the serum of quails in different experimental stages. **(A)** The expression levels of IL-1β in the serum. **(B)** The expression levels of TNF-α in the serum. **(C)** The expression levels of IL-6 in the serum. **(D)** The expression levels of IL-8 in the serum. **(E)** The expression levels of IL-18 in the serum. Data are expressed as mean ± SEM for 8 quails in each group. *P < 0.05, **P < 0.01 and ***P < 0.001 versus Con group.

ELISA detection kits were used to detect the changes in cytokines such as IL-1β, IL-6, IL-18, TNF-α, and IL-8. The results showed that compared with the Con group, the expression levels of TNF-α and IL-8 cytokines in the quails of the Mod group at 10 days, 20 days, and 30 days were significantly increased (*P*< 0.05 or *P*< 0.01) (**Figures 4B, D**). The expression levels of IL-1β, IL-6, and IL-18 cytokines in the quails of the Mod group were significantly higher than those of the Con group on the 20th and 30th days of the experiment (*P*<0.05 or *P*<0.01) (**Figures 4A, C, E**).

### The uric acid level in synovial fluid, polarized light observation, and HE staining

**Figure 5:**
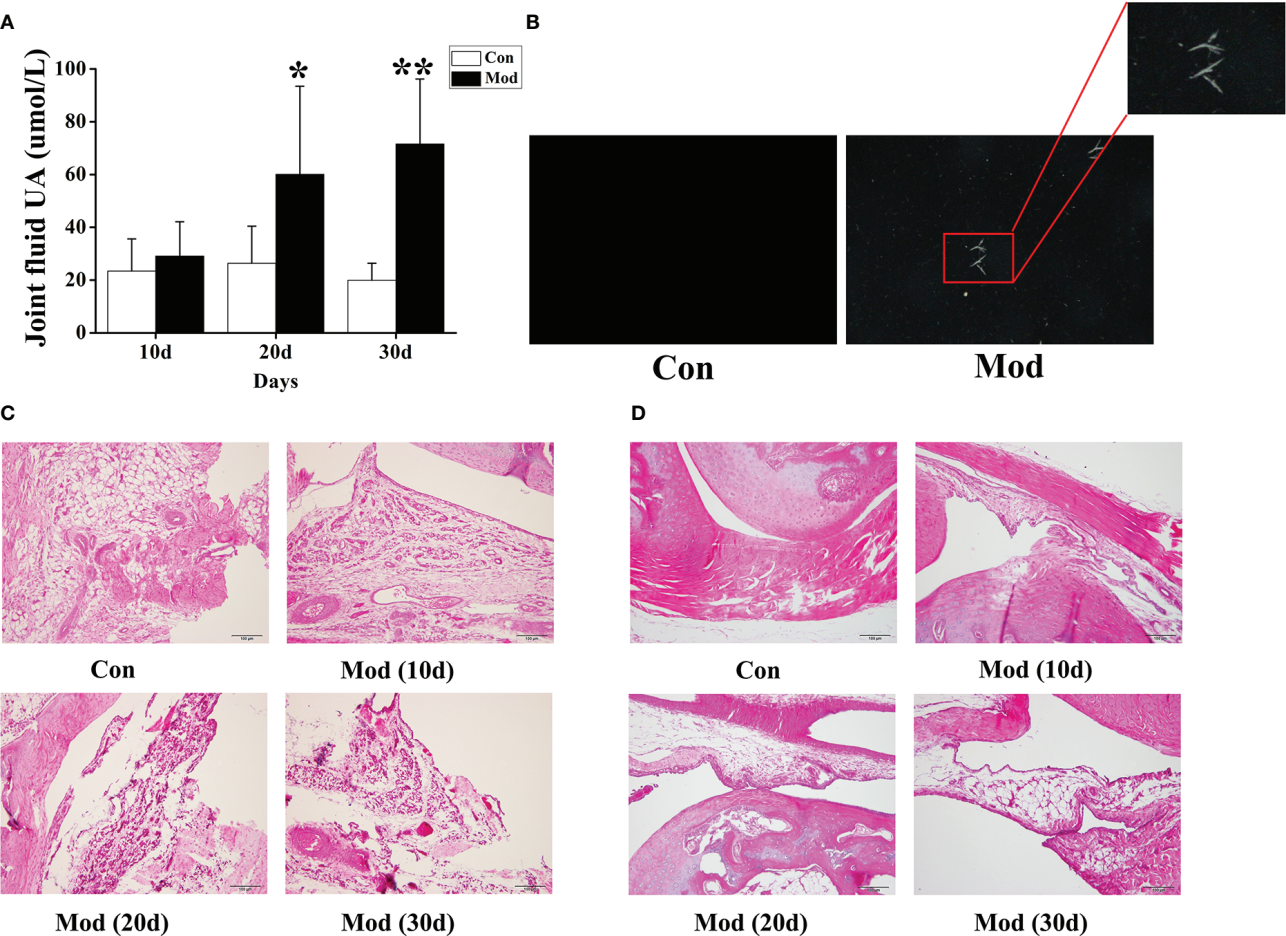
The levels of uric acid in joint fluid and HE staining of joints of quails in different experimental stages. **(A)** The joint fluid uric acid level of the quails. **(B)** The urate crystals appeared in the synovial fluid of quails. **(C)** HE staining of ankle joint (200×). **(D)** HE staining of claw joint (200×). Data are expressed as mean ± SEM for 8 quails in each group. *P < 0.05 and **P < 0.01 versus Con group.

Compared with the Con group, the synovial fluid uric acid level of the quails in the Mod group was significantly increased at 20 and 30 days (*P*< 0.05 or *P*< 0.01) (**Figure 5A**). Taken 10ul of synovial fluid, fixed it on a glass slide, and observed it under a polarizing microscope. No urate crystals were found in the Con group. Compared with the Con group, many blue-yellow bright, needle-shaped, or thin rod-shaped urate crystals were seen in the Mod group. The results showed that urate crystals appeared in the synovial fluid of all quails in the Mod group at 30 days (**Figure 5B**).

The ankle joints and claw joints of quails in each group were stained with HE. The HE staining (200×) of the ankle joints showed that the quails in the Con group had a clear structure of the ankle joints, the joint surface was smooth, and the synovial tissue morphology was expected; the quails in the Mod group increased with the modeling time. In the ankle joint tissue, synovial tissue erodes the cartilage surface, and synovial fibrous tissue hyperplasia is accompanied by a large number of inflammatory cell infiltration and surrounding tissue edema (**Figure 5C**). HE was staining of quail claw joint tissue showed that the quail claw common tissue structure of the Con group was clear, and the synovial tissue morphology was complete. With the increase of modeling time in the Mod group, many inflammatory cell infiltration and synovial fibrous tissue hyperplasia were found in the claw joint tissue (**Figure 5D**).

### Oxidative stress levels

**Figure 6:**
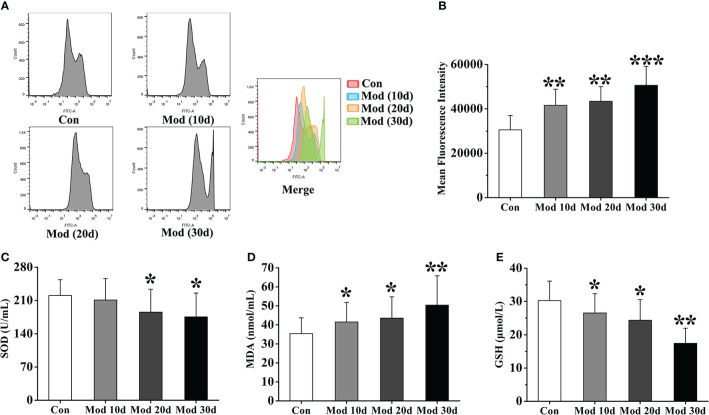
The level of oxidative stress in the joint synovial tissue of quails at different experimental stages. **(A)** Detection of ROS expression levels in the joint synovial tissue by flow cytometry. **(B)** The mean fluorescence intensity of the joint synovial tissue. in each group. **(C)** The content of SOD in the joint synovial tissue. **(D)** The content of MDA in the joint synovial tissue. **(E)** The content of GSH in the joint synovial tissue. Data are expressed as mean ± SEM for 8 quails in each group. *P < 0.05, **P < 0.01 and ***P < 0.001 versus Con group.

Flow cytometry detected ROS expression in each group’s joint synovial tissue. The experimental results showed that compared with the Con group, the ROS fluorescence intensity of the Mod group was significantly increased (*P*<0.01 or *P*<0.001), indicating that with the increase of modeling time, the expression of ROS in synovial tissue of quail in Mod group also increased continuously (**Figures 6A, B**). We detected the level of oxidative stress in the joint synovial tissue of quails at different stages. The results showed that on the 10th day of the experiment, the content of MDA in the Mod group was significantly higher than that of the Con group (*P*<0.05), and the GSH content was significantly lower than that of the Con group (*P*<0.05). On the 20th day, the contents of SOD and GSH in the joint synovial tissue of quails in the Mod group were significantly lower than those in the Con group (*P*< 0.05), and the contents of MDA and GSH were significantly higher than those in the Con group (*P*< 0.05). On the 30th day, the MDA level in the Mod group was significantly higher than that in the Con group (*P*< 0.01), and the SOD and GSH contents were significantly lower than those in the Con group (*P*< 0.05 or *P*< 0.01) (**Figures 6C–E**).

### Effects of adding allopurinol on gouty quail

**Figure 7:**
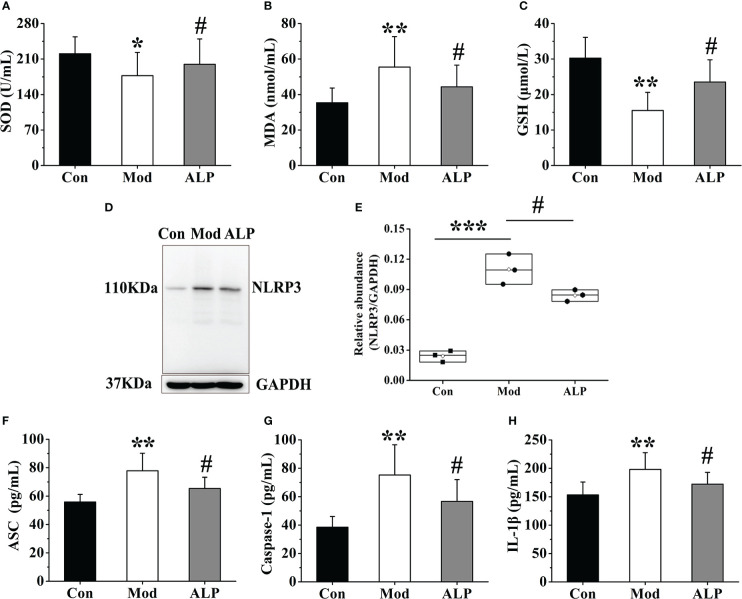
Allopurinol alleviates oxidative stress and NLRP3 inflammasome in gouty quail. **(A)** The content of SOD in the joint synovial tissue. **(B)** The content of MDA in the joint synovial tissue. **(C)** The content of GSH in the joint synovial tissue. **(D)** Western blot was conducted to evaluate the protein level of NLRP3 and GAPDH in synovial tissue. **(E)** Box plot showing the densitometry analysis of NLRP3 normalized by GAPDH. **(F)** The expression level of ASC in synovial tissue. **(G)** The expression level of Caspase-1 in synovial tissue. **(H)** The expression level of IL-1β in synovial tissue. Data are expressed as mean ± SEM for 8 quails in each group. *P < 0.05, **P < 0.01 and ***P < 0.001 versus Con group. ^#^ *P* < 0.05 versus Mod group.

To further clarify the role of XOD in oxidative stress and NLRP3 inflammasome activation, allopurinol, an inhibitor of XOD, was selected as the experimental drug for verification. On the 30th day of the experiment, ten quails in the Mod group were chosen for allopurinol gavage treatment. After 10 days of administration, the expressions of SOD, MDA, and GSH in the joint synovial tissue were detected. And the synovial tissue of the quails in each group was separated to detect the expression of NLRP3 protein and ASC, Caspase-1, and IL-1β in synovial tissue homogenate. The results showed that the expression levels of SOD and GSH in the Mod group were significantly lower than those of the Con group (*P*<0.05 or *P*<0.01), and the expression level of MDA was significantly higher than that of the Con group (*P*<0.01). The expression levels of SOD and GSH of the ALP group were significantly increased compared with the Mod group (*P*<0.05), and the expression level of MDA was significantly lower than that in the Mod group (*P*<0.05), indicating that allopurinol can alleviate oxidative stress by inhibiting XOD (**Figures 7A–C**). After analyzing the protein expression of NLRP3 in the quail synovial tissue of each group, the results showed that the expression of NLRP3 in the Mod group was significantly higher than that in the Con group (*P*<0.001), and the expression of NLRP3 in the ALP group was significantly lower than that in the Mod group (*P*<0.001) (**Figures 7D, E**). The expression levels of ASC, Caspase-1, and IL-1β in each group’s synovial tissue of quails were analyzed. The expression levels of ASC, Caspase-1, and IL-1β in the Mod group were significantly higher than in the Con group (*P*<0.01). After allopurinol treatment, the results showed that the expression levels of ASC, Caspase-1, and I, L-1β in the ALP group were significantly lower than those in the Mod group (*P*<0.05) (**Figures 7F–H**).

### Extraction and identification of primary fibroblast-like synoviocytes from quail

**Figure 8:**
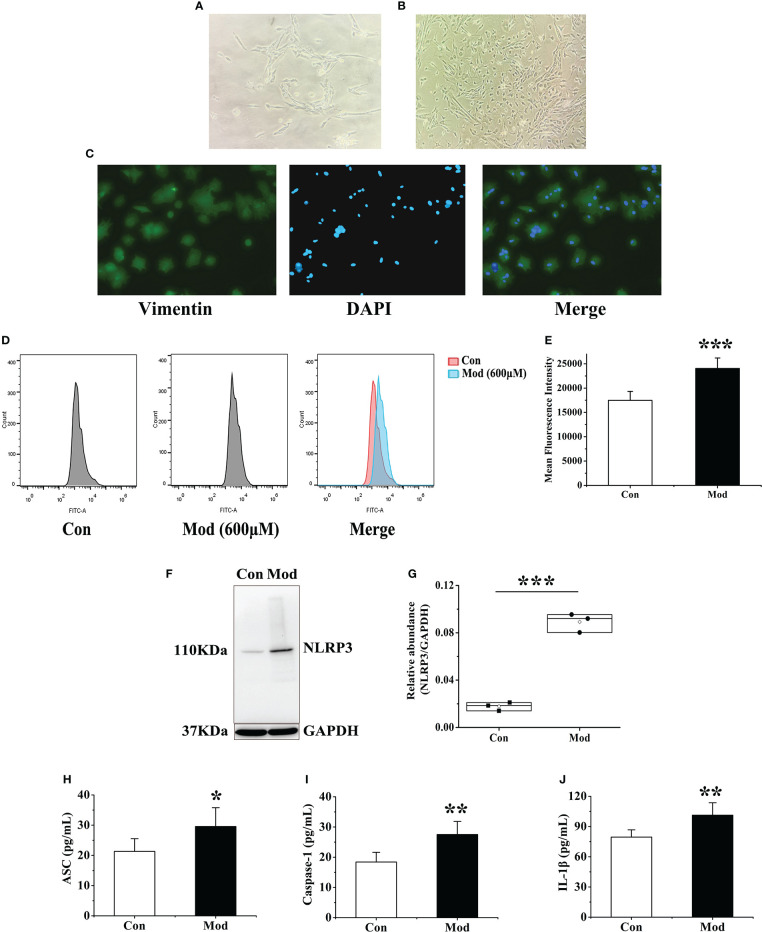
Extraction and identification of FLS and its effect on cellular ROS and NLRP3 inflammasome after uric acid treatment. **(A)** Morphology of the first generation FLS. **(B)** Morphology of the third-generation FLS. **(C)** Vimentin identifies FLS as a characteristic labeling protein. **(D)** Detection of ROS expression levels in FLS after adding uric acid by flow cytometry. **(E)** The mean fluorescence intensity of FLS in each group. **(F)** Western blot was conducted to evaluate the protein level of NLRP3 and GAPDH in cell lysates. **(G)** Box plot showing the densitometry analysis of NLRP3 normalized by GAPDH. **(H)** The expression level of ASC in cell supernatant. **(I)** The expression level of Caspase-1 in cell supernatant. **(J)** The expression level of IL-1β in cell supernatant. *P < 0.05, **P < 0.01 and ***P < 0.001 versus Con group.

The isolated synovial tissue began to adhere to the wall after 6-8 hours of culture. After one day, the cells migrated out, and most of them were spindle-shaped (**Figure 8A**). On the 5th day of culture, the cell confluence reached 70% to 80% adherent growth. On the 12th day of culture, the synovial cells passed to the third passage were all spindle-shaped. Synovial cells in passages 3-7 were proliferating and growing well (**Figure 8B**). The third generation of synovial cells obtained by the collagenase digestion method was observed by immunostaining: the morphology was regular and spindle-shaped, and its nucleus was oval and located in the center of the cell. The positive rate of vimentin staining of synovial cells was greater than 98% (**Figure 8C**). Vimentin is a characteristic labeling protein of FLS, suggesting that the cultured synovial cells are FLS, and the purity is more than 98%.

### Effects of uric acid stimulation on the expression of ROS and NLRP3 inflammasome in FLS

According to the previous research of the research group and the results of cell viability experiments, 600 μM exogenous uric acid was used to stimulate FLS as the Mod group. After 24 hours, flow cytometry was used to detect the expression of ROS in the supernatant of each group. The experimental results showed that compared with the Con group, the ROS fluorescence intensity of the Mod group was significantly increased (*P*<0.001), indicating that uric acid stimulation made synovial cells in a state of oxidative stress (**Figures 8D, E**). The expressions of NLRP3, ASC, Caspase-1, and IL-1β in the cell lysates of each group were detected, and the results showed that the expression level of NLRP3 protein in the Mod group was significantly higher than that in the Con group (*P*<0.001) (**Figures 8F, G**). After stimulation with source uric acid, the results showed that the expression levels of ASC, Caspase-1, and IL-1β in the Mod group were significantly higher than those in the Con group (*P*<0.05 or *P*<0.01) (**Figures 8H–J**).

### Effects of inhibition of XOD expression on ROS and NLRP3 inflammasome in FLS

**Figure 9:**
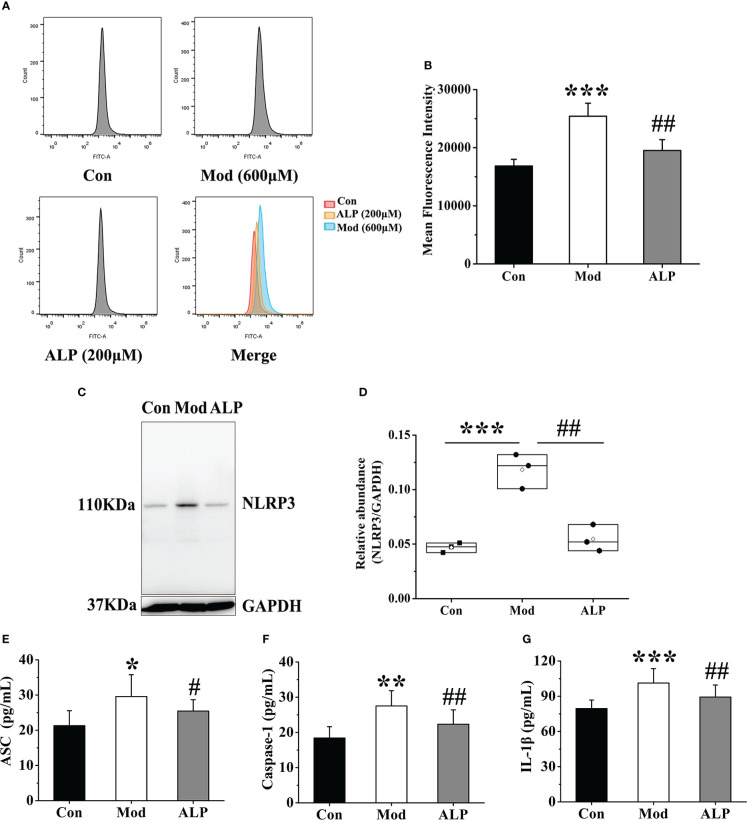
Allopurinol inhibits ROS and NLRP3 inflammasome in FLS after uric acid stimulation. **(A)** Detection of ROS expression levels in FLS by flow cytometry. **(B)** The mean fluorescence intensity of FLS in each group. **(C)** Western blot was conducted to evaluate the protein level of NLRP3 and GAPDH in cell lysates. **(D)** Box plot showing the densitometry analysis of NLRP3 normalized by GAPDH. **(E)** The expression level of ASC in cell supernatant. **(F)** The expression level of Caspase-1 in cell supernatant. **(G)** The expression level of IL-1β in cell supernatant. *P < 0.05, **P < 0.01 and ***P < 0.001 versus Con group. ^#^ *P* < 0.05 and ^##^ *P* < 0.01 versus Mod group.

To further explore the activation of ROS and NLRP3 inflammasome by FLS through XOD, the XOD inhibitor allopurinol was selected for verification. According to the previous research of the research group and the results of cell viability experiments, 200 μM allopurinol was chosen as the experimental drug group for verification. The Mod group was stimulated with 600 μM uric acid, while the ALP group was produced with uric acid and added 200 μM allopurinol was for observation. After 24 hours, flow cytometry was used to detect the expression of ROS in the supernatant of cells in each group. Compared with other groups, the fluorescence intensity of ROS in the Mod group was significantly increased (*P*<0.001). In contrast, the fluorescence intensity of ROS in the ALP group was significantly lower than that in the Mod group (P<0.01), indicating that uric acid stimulation increased the expression of ROS in FLS. At the same time, allopurinol can reduce ROS expression by inhibiting XOD (**Figures 9A, B**).

The expressions of NLRP3, ASC, Caspase-1 and IL-1β in the cell lysates of each group were further detected. The results showed that the expression level of NLRP3 protein in the Mod group was significantly higher than in the Con group (P<0.001). The expression level of NLRP3 protein in the ALP group was significantly lower than that in the Mod group (*P*<0.01) (**Figures 9C, D**). At the same time, the expression levels of ASC, Caspase-1, and IL-1β in the Mod group were significantly higher than those in the Con group (*P*<0.05 or *P*<0.01 or *P*<0.001). In contrast, the ALP group had a higher expression level of ASC, Caspase-1, and IL-1β, significantly lower than the Mod group (*P*<0.05) (**Figures 9E–G**).

## Discussion

With the change in people’s living standards and dietary structure, the incidence of gout has an evident trend of increasing, and it is one of the metabolic diseases that seriously threaten the physical and mental health of human beings (1, 41). Recent studies suggest that gouty arthritis and hyperuricemia are different periods of the same disease (10). The core of gout is the uric acid and deposition of urate, which induces an inflammatory response. Existing studies have also found that inflammatory factors are elevated in patients with asymptomatic hyperuricemia, which indicates that an inflammatory reaction occurs in the stage of hyperuricemia (42–45). Recent studies have confirmed that the NLRP3 inflammasome is activated during the onset of gouty arthritis. However, the effect of NLRP3 inflammasome on gout’s overall pathological process is unclear. And what causes the activation of the NLRP3 inflammasome is partially transparent in gout. In this study, we found that the expression of uric acid-producing enzyme XOD, the expression level of ROS, and the activation of NLRP3 inflammasome were increased in the pathogenesis of gouty quail.

This study innovatively established a gout quail model from hyperuricemia to gouty arthritis, which can provide an experimental carrier for researching and developing gout prevention and treatment drugs. Our research group has been engaged in the study of gout-related animal models for a long time. We found that in the animal model of gout, Coderre et al. successfully established the animal model of gout arthritis by local joint injection of sodium urate (46). A large number of subsequent scholars adopted this classical modeling method. In the simulation of model animals, knee joint injection, ankle joint injection, and toe pad injection can be seen. However, this method can only reflect local joint symptoms and is suitable for observing common symptoms. It only causes a temporary non-specific inflammatory reaction and cannot form a lasting model. In this study, quails with similar uric acid metabolism pathways to humans were used as model animals, which had physiological consistency with human uric acid metabolism. They realized the complete pathological process of gout disease, from high blood uric acid to urate deposition and joint inflammation. In addition, the model method simulated the etiology of clinical gout disease. A high purine diet and fructose were used as inducers to promote uric acid production and inflammation in the body. The results indicated that the quail model could be used as an excellent animal model for studying pathological characteristics and mechanisms of gout.

Hyperuricemia is the biochemical basis of gout, and its pathological process is related to the target of uric acid metabolism. Recent studies have found that uric acid metabolic targets are associated with inflammatory responses and regulating uric acid levels (22, 23). XOD is the enzyme responsible for uric acid production in the target metabolism group. Serum IL-1β levels are significantly reduced in hyperuricemic patients when an XOD inhibitor is used to lower uric acid (29). When uric acid-lowering drugs are administered to treat hyperuricemia, a decrease in inflammatory factors in patients can be detected (28). The essence of its inflammation may be that the changes in uric acid metabolism targets are involved in forming urate deposition and activating the inflammasome. During the experiment, the renal function of the quails in each group was detected, and the kidneys were stained with HE. The results showed that the quail kidneys in the Mod group had inflammatory damage (**Figure S1**). The results of this study also confirmed that XOD plays an essential role in the development of gout, which can elevate uric acid levels, promote the release of ROS and activate the NLRP3 inflammasome.

Uric acid is the end product of purine metabolism in the body. The average physiological concentration of uric acid is an essential antioxidant in the body, which can reduce oxidative damage caused by ROS. Higher than physiological uric acid concentrations can induce mitochondrial damage and promote ROS release (46). During uric acid generation, the xanthine oxidase system produces oxygen free radicals, one of the body’s primary sources of free radicals (47, 48). The central role of xanthine oxidase in various pathological conditions is related to its oxidation of xanthine and hypoxanthine to release superoxide anion free radicals and hydrogen peroxide. The results showed that the XOD activity of gouty quail was significantly increased (*P*<0.05). SOD is the most important free radical in the body, as a natural scavenger of superoxide radicals. It catalyzes superoxide-free radical disproportionation, reduces MDA content, and minimizes harm to the human body. This study found that with the increase in XOD expression, the levels of SOD and GSH in the Mod group were significantly decreased, and the expression level of MDA increased considerably. After allopurinol treatment, the body’s degree of peroxidation damage and antioxidant ability was recovered. Meanwhile, many studies have shown that increased XOD activity can release oxygen free radicals and further promote ROS expression. The results of this study indicate that XOD inhibitors can not only produce ROS *in vivo* but also inhibit ROS produced by FLS stimulated by uric acid *in vitro*. This suggests that XOD may be an essential target affecting oxidative stress balance *in vivo*.

Recent studies have shown that the activated NLRP3 inflammasome is closely associated with the inflammatory response in gout (49, 50). When the NLRP3 inflammasome is overactivated, many inflammatory factors are formed, triggering an inflammatory response *in vivo* and promoting gout development (51). The activation pathways of NLRP3 mainly include the ion current-mediated pathway, lysosomal destruction pathway, and ROS-mediated pathway. ROS levels are closely related to the activation of the NLRP3 inflammasome. Reducing ROS production can significantly inhibit the activation of the NLRP3 inflammasome, and vice versa can promote its activation (52–54). The results of this study suggest that when XOD expression is elevated, disrupts the balance of oxidative stress, and generates a large amount of ROS, which further activates the NLRP3 inflammasome and subsequently releases matured pro-inflammatory cytokines, including IL-1β. As a core cytokine of gout inflammation, IL-1β can stimulate the expression of other inflammatory factors through autocrine and paracrine ways, promote the inflammatory cascade and participate in the development of gout inflammation (55, 56).

Our data showed that the increase in uric acid level was correlated with XOD activity, and the expression of XOD in the Mod group was always higher than that in the Con group at different stages of the experiment (P<0.05). The activation of NLRP3 inflammasome accompanies the onset of gouty arthritis in quail. By observing the whole pathological process of gout, we found that the relationship between the uric acid metabolism target XOD and the NLRP3 inflammasome may be the critical immune response between hyperuricemia and gout. Our data showed that, in the process of gout disease, the expression of XOD increases, which increases the level of uric acid in the body, disrupts the balance of oxidative stress, generates a large amount of ROS, activates the NLRP3 inflammasome, and release IL-1β. Meanwhile, many studies have shown that increased XOD activity can release oxygen free radicals and further promote ROS expression. The results of this study indicate that XOD inhibitors can not only produce ROS *in vivo* but also inhibit ROS produced by FLS stimulated by uric acid *in vitro*. This study is the first to clarify the changes in critical pathological targets during the whole pathological process of gout and to explore the correlation between uric acid levels and inflammatory expression. Our results indicate that the immune response between the uric acid metabolism target XOD and NLRP3 inflammasomes plays a crucial role in developing hyperuricemia to gout, and inhibition of both XOD and NLRP3 inflammasomes may be an effective treatment for avoiding the development of asymptomatic hyperuricemia to MSU crystal deposition.

Collectively, this study confirms that the increased XOD activity, ROS expression, and NLRP3 inflammasome play an essential role in inflammasome dysregulation and triggers gout. In the pathological process of gout, the increased expression of XOD can promote the production of uric acid, disrupt the balance of oxidative stress, generate a large amount of ROS, activate the NLRP3 inflammasome, and release IL-1β. Our study extracted and identified primary synovial fibroblasts in quail and applied them to the mechanism of research in gout. In subsequent studies, we will screen for new inflammatory signaling pathways and urate-lowering target proteins in FLS by transcriptome sequencing. And it will provide a new reference for the study of gout. The quail model of gout used in this study is consistent with human uric acid metabolism, simulates clinical etiology, and fits the disease progression, which can provide an animal model for the study of the pathological characteristics and mechanism of gout. At the same time, this study clarified the critical role and correlation of uric acid metabolism targets and NLRP3 inflammasome in the progression of gout, which can provide a reference for clinical drug development and efficacy evaluation of gout prevention and treatment.

## Data availability statement

The original contributions presented in the study are included in the article/**Supplementary Material**. Further inquiries can be directed to the corresponding author.

## Ethics statement

The animal study was reviewed and approved by NO. BUCM-4-2021070102-3105.

## Author contributions

BZ and ZL conceived the study. HW wrote the manuscript. YW, ZR, YL, and JH reviewed and revised the manuscript. All authors contributed to the manuscript revision and read and approved the submitted version.
