# Supplementary material for: Overnutrition-induced gout: An immune response to NLRP3 inflammasome dysregulation by XOD activity increased in quail
Source: Front Immunol. 2022 Dec 8;13:1074867. doi: 10.3389/fimmu.2022.1074867 (PMC9771704; doi:10.3389/fimmu.2022.1074867)
Supplement: Supplementary file 1 [file DataSheet_1.docx]

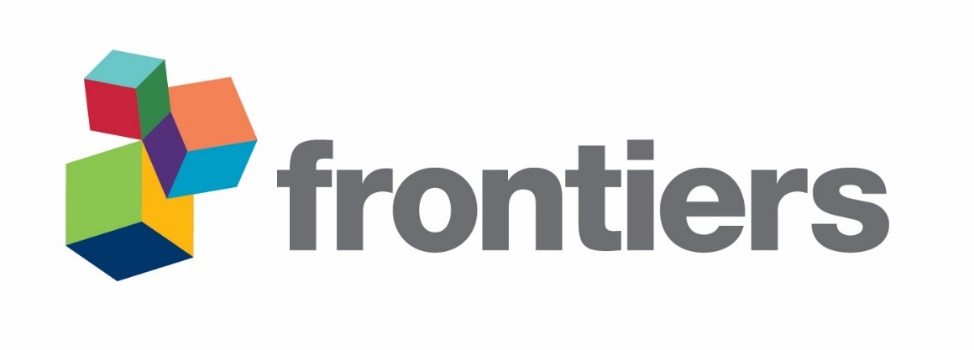
Supplementary Materia

**Renal function level and staining of HE**

The renal function level of quails in each group was detected during the experiment. Compared with the Con group, the serum urea nitrogen level of the quails in the Mod group was significantly increased on the 10th, 20th, and 30th days of the experiment (*P*<0.05 or *P*<0.01) **(Figure S1-A)**. Compared with the Con group, on the 30th day of the experiment, the creatinine level of the quails in the Mod group was significantly decreased (*P* < 0.05 or *P* < 0.01). Compared with the Con group, the kidney index of the quails in the Mod group was significantly increased on the 30th day of the experiment (*P* < 0.05 or *P* < 0.01). Compared with the Con group, the fractional renal uric acid excretion of the quails in the Mod group was significantly lower at 20 and 30 days of the experiment (*P* < 0.05 or *P* < 0.01) **(Figure S1-BCD)**.

The HE staining of quail kidney tissue showed that the structure of the quail kidney in the Con group was normal, and the quail kidney tissue in the Mod group showed a large number of glomerular atrophy, tubular lumen expansion, and necrosis and shedding of epithelial cells. And with the prolongation of modeling time, kidney damage was further aggravated **(Figure S1-E)**.

**
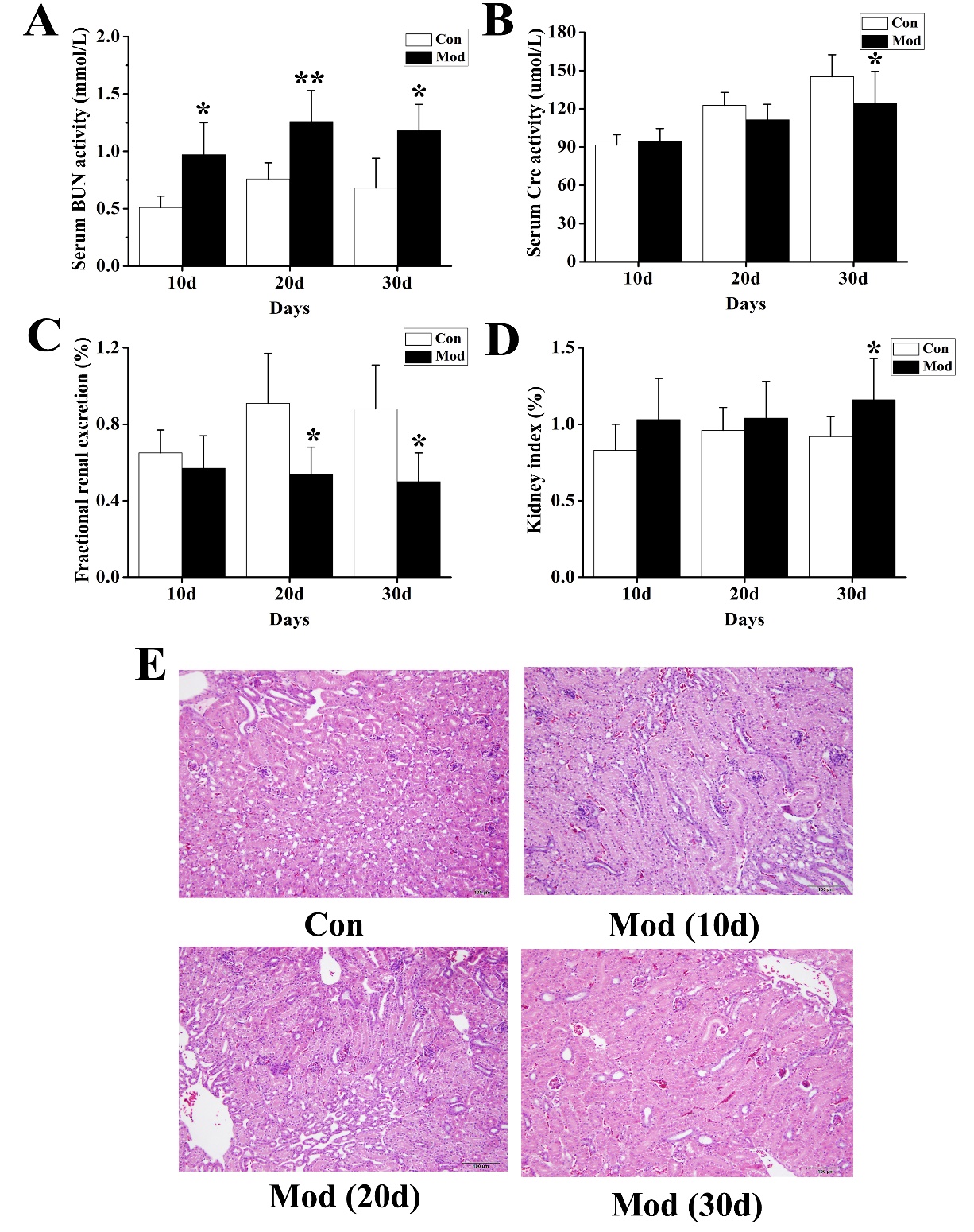
**

**Figure S1：**Serum kidney function, HE staining, and urate staining of quails in different groups. (A)

The activity of BUN in the serum. (B) The activity of Cre in the serum. (C) The fractional renal uric acid excretion of the quails. (D) Kidney index. (E) HE staining. **P* < 0.05, ***P* < 0.01 and ****P* < 0.001 versus Con group.
